# Loneliness and low fruit and vegetable consumption among adults in Japan

**DOI:** 10.3389/fpubh.2024.1365628

**Published:** 2024-10-28

**Authors:** Andrew Stickley, Aya Shirama, Tomiki Sumiyoshi

**Affiliations:** Department of Preventive Intervention for Psychiatric Disorders, National Institute of Mental Health, National Center of Neurology and Psychiatry, Kodaira, Japan

**Keywords:** lonely, fruits, vegetables, Japanese, public health

## Abstract

**Background:**

Loneliness is the distressing feeling that arises when a person's network of social relations is perceived as being inadequate in some way. Research has linked loneliness to a number of detrimental health outcomes. There is also some evidence that lonely individuals are more likely to engage in poorer health behaviors. However, as yet, there has been comparatively little attention paid to the relation between loneliness and dietary behavior. In particular, there has been little focus on the association between loneliness and fruit and vegetable intake.

**Objective:**

The aim of this cross-sectional study was to examine the association between loneliness and low fruit and vegetable consumption in the Japanese general population.

**Methods:**

Data were analyzed from 3,410 Japanese adults collected in an online survey in early 2023. Information was obtained on past-week fruit and vegetable consumption with a single-item measure, while loneliness was assessed with the Three-Item Loneliness Scale. Information was also collected on sociodemographic characteristics, physical health status, health-risk behaviors and depressive symptoms. Logistic regression was used to assess associations.

**Results:**

One in twenty (5.3%) adults reported low fruit and vegetable consumption. In a fully adjusted analysis loneliness was associated with higher odds for low fruit and vegetable consumption (OR: 1.14, 95%CI: 1.04–1.26). In sex- and age-stratified analyses loneliness was significantly associated with low fruit and vegetable consumption in both women and middle-aged adults, although confidence intervals overlapped for this association across all of the sex and age groups in the fully adjusted analyses.

**Conclusions:**

Loneliness is associated with low fruit and vegetable consumption among adults in Japan. As loneliness and inadequate fruit and vegetable intake have both been linked to poorer health outcomes, the results of this study underscore the potential importance and public health benefits of reducing loneliness in Japan.

## 1 Introduction

Loneliness is the unpleasant experience that occurs as a result of a deficiency in the quantity or quality of one's network of social relations (1). A recent systematic review and meta-analysis that used data from 113 countries concluded that problematic levels of loneliness are observed in many countries (2), while there is also some evidence that the prevalence of loneliness may have further increased across sex and age groups during the COVID-19 pandemic (3). The presence of high and increasing levels of loneliness is worrying, as although for many people the experience of loneliness is transient and thus considered normative, for others loneliness can be a chronic phenomenon (4) that can have detrimental consequences for health (5). In particular, studies have linked loneliness to poorer physical and mental health outcomes including depression (6), cardiovascular disease and diabetes (7), stroke and pulmonary problems (8), physical multimorbidity (9), and even an increased risk of mortality (10). It is possible that various mechanisms might link loneliness and poorer health. Some research has indicated for example, that lonely individuals may be exposed to more stressors and differ in their appraisals of stress (11). Health behaviors might also be important in this context as loneliness has been linked to physical inactivity (12) and smoking (13).

This study will examine the association between loneliness and one type of health behavior – dietary intake – in Japanese adults. More specifically, we will examine the relation between loneliness and low fruit and vegetable consumption. Research has shown that there is an insufficient availability of fruit and vegetables in many countries across the world (14), which may partly explain the high prevalence of fruit and vegetable intake below recommended dietary guidelines in both low- and high-income countries (15–17). As low fruit and vegetable intake may also be linked to worse physical and mental health (18), then understanding its association with loneliness may have important public health implications. However, as yet, there have been comparatively few studies that have examined this association in either the general population or specific population subgroups. In terms of the former there have been three studies and these have produced mixed results. A study from Switzerland that used data from people in the general population aged 15 and above found that lonely individuals were less likely to adhere to fruit and vegetable consumption guidelines (19). In contrast, a study that used national data from Indonesian participants aged 15 and above found that loneliness was not significantly associated with ‘infrequent' fruit (i.e. less than 3 days a week) and vegetable consumption (i.e. less than daily) in a fully adjusted logistic regression analysis (20). In another study among adults from the Spanish city of Alcalá de Henares in Madrid, Spain, in an analysis restricted to adults aged 30-44 who were living with other people, loneliness was not associated with daily fruit or vegetable consumption in either men or women (21). Similar mixed results have been seen in studies among population subgroups. For example, in a study among residents from deprived neighborhoods in Denmark, loneliness was associated with a low intake of fruit and vegetables (i.e. not during a week) (22), while in another study among adults living with and beyond cancer (LWBC) higher levels of loneliness were linked to lower odds for meeting World Cancer Research Fund fruit and vegetable intake recommendations (23). In contrast, in a longitudinal study among English adults aged 52 and above, loneliness was not associated with the consistent consumption of ≥5 daily servings of fruit and vegetables over a 10-year period (24). In summary, to date, research among both the general population and population subgroups has produced mixed results. The categorization of both fruit and vegetable consumption and loneliness has varied between studies with this latter phenomenon being variously assessed with single-item questions (19, 20), the Three-Item Loneliness Scale (23–25) and the longer 20-item UCLA Loneliness Scale (version 3) (26). With some exceptions (20, 26, 27) most research has been undertaken in Western countries and there has been almost no research attempting to determine if there are sex (21) or age differences in this association.

In line with other research that has been undertaken on aspects of mental health, wellbeing and food intake during the COVID-19 pandemic (28), examining the association between loneliness and low fruit and vegetable consumption in Japan at the tail end of the pandemic might be instructive. A recent study found that the prevalence of loneliness was high in the general population in Japan during the COVID-19 pandemic (29), while earlier research indicated that many lonely Japanese adults may experience chronic feelings of loneliness (lasting more than 10 years) (30). Importantly, fruit and vegetable consumption has also declined in Japan in recent decades, with the consumption of vegetables constituting only 80% of the government's daily recommended target in 2019 (31), possibly because of the comparatively high cost of these food items (32), although there is some indication that the purchasing of fruit and vegetables may have increased during the coronavirus pandemic – possible driven by increased health awareness (33). As yet, there has been little focus on the association between loneliness and fruit and vegetable consumption in Japan. Specifically, although one study found that loneliness was linked to a lower frequency of fruit and vegetable consumption, it used data from only 57 adults and was restricted to those aged 60 and above (27).

This study therefore has two aims. Firstly, to examine if loneliness is associated with low fruit and vegetable consumption among adults in the Japanese general population. Second, as there is some evidence that there are sex and age differences in low fruit and vegetable intake (34), including in Japan (31), while a recent study from China found that loneliness was associated with gender differences in unhealthy dietary behavior (that included having an unbalanced diet due to insufficient fruit and vegetable consumption) (35), we will also examine if there are sex and/or age differences in the association between loneliness and low fruit and vegetable intake.

## 2 Methods

### 2.1 Study participants

Data were collected from an online survey of the Japanese general population undertaken in March 2023. The survey was administered by a Japanese market research company specializing in the healthcare sector. In the first phase of the survey, based on previous selections, the company contacted 22,991 randomly selected individuals from its large online web panel (which is representative of the Japanese general population) about participating in the survey. Individuals were selected using a 16-cell system selection procedure (i.e. eight age cells by two sex cells) which is designed to reflect the composition ratio of the Japanese population, where the cells were automatically closed when the selection target for a specific age-sex combination was obtained. At the end of the selection procedure and in line with previous studies (36, 37), this generated a final sample of 3,717 participants, where all of those individuals who were selected and agreed to participate were aged 18 years old and above, where the sex distribution was representative of the Japanese population and where respondents were drawn from each of Japan's regions (i.e. the 47 prefectures). Permission for the survey was obtained from the ethics committee at the National Center of Neurology and Psychiatry, Tokyo (approval number: A2022-096). All participants provided informed consent.

### 2.2 Measures

#### 2.2.1 Low fruit and vegetable consumption

Fruit and vegetable consumption was assessed with a question that asked, “How often in the past week have you eaten (1) Fresh vegetables (not including potatoes); (2) Fresh fruit” with the response options, (i) daily/almost daily; (ii) several times per week; (iii) once a week; (iv) less than once a week; (v) don't know. In line with several previous studies that have used less than weekly as a cut-off point (38, 39), in this study those respondents who stated that they ate both fruit and vegetables less than once in the week were categorized as having low fruit and vegetable consumption.

#### 2.2.2 Loneliness

This was assessed with the Three-Item Loneliness Scale (40), which enquires about a lack of companionship, feeling left out and feeling isolated. The total scale score ranges from 3 to 9 with higher scores indicating increased loneliness. This measure has been validated for use in the Japanese population (41) and has been previously used in Japan to assess loneliness (29). Cronbach's alpha for the scale was 0.76. The score was used as a continuous variable in the analysis.

#### 2.2.3 Covariates

Information was obtained on sex (male, female) and age, categorized as 18-34, 35-59 and ≥ 60. In terms of education respondents were categorized as having either a (i) higher education (two-year college, university, graduate school), or (ii) less than a higher education (junior high school, high school, specialized vocational high school). For marital status respondents were categorized as being either (i) married/cohabiting; (ii) never married (single); or (iii) divorced/widowed. Four categories were used for household income, which was measured in millions of yen: (i) <4 million; (ii) 4 <10 million; (iii) ≥ 10 million; (iv) ‘missing' (as 21.8% of the analytic sample did not provide information for this variable) (132.93 JPY = 1 USD at the time of the survey). Information was also collected (with yes/no answer options) on eight medical conditions: (i) high blood pressure, (ii) stroke (e.g. brain hemorrhage, cerebral infarction), (iii) heart disease, (iv) diabetes, (v) respiratory disease (e.g. pneumonia, bronchitis), (vi) gastrointestinal, liver, or gallbladder disease, (vii) kidney or prostate gland disease, (viii) cancer (malignant tumor). The number of conditions was summed and then a three-category variable was created: 0, 1, ≥ 2 medical conditions. Respondents were also asked to provide self-reports of their weight (in kg) and height (in cm). This information was used to create a Body Mass Index (BMI) variable with four categories: 18.5-24.9 (standard weight), <18.5 (underweight), 25.0-29.9 (overweight), ≥ 30 (obese). The four-item CAGE questionnaire (42), which enquires about different aspects of drinking difficulty was used to assess problematic alcohol use. The total scale score can range from 0 to 4 with higher scores indicating greater problems. In the current study a score of ≥ 2, which is considered clinically significant (43), was used to categorize cases of problematic alcohol use. Cronbach's alpha was 0.68 for the scale. For smoking status participants were categorized as never smokers, former smokers, or current smokers. Finally, past two-week depressive symptoms were assessed with the nine-item Patient Health Questionnaire (PHQ-9) (44). The total scale score ranges from 0 to 27 with higher scores indicating more depressive symptoms. In this study a score of ≥ 10 was used to categorize at least a moderate level of depressive symptoms (45) (Cronbach's alpha was 0.89 for this measure).

### 2.3 Statistical analysis

Descriptive statistics of the study sample by low fruit and vegetable consumption status were first calculated, with Chi-square and Mann Whitney U tests used to examine differences between the categories and continuous scores, respectively. Next, logistic regression was used to examine the association between loneliness and low fruit and vegetable consumption. In relation to this, as previous research has indicated that a variety of variables are linked to both fruit and vegetable consumption and loneliness including sociodemographic factors (46–49), physical health status (19, 47, 50), health-risk behaviors (19, 46, 47, 51) and mental health (19, 48, 52), a hierarchical analysis was undertaken where each of these variable groups was entered into the analysis in subsequent models to determine if they affected the association between loneliness and low fruit and vegetable consumption. Thus, 5 models were used in the analysis. In Model 1 the bivariate association between loneliness and low fruit and vegetable consumption was examined. Model 2 was additionally adjusted for the sociodemographic variables – sex, age, education, marital status and household income. Model 3 included the same variables as in Model 2 and was additionally adjusted for physical health status i.e., medical conditions and BMI. Model 4 included the same variables as in Model 3 and was additionally adjusted for two health-risk behaviors—smoking and problematic alcohol use. The fully adjusted Model 5 included the same variables as Model 4 and was additionally adjusted for mental health (depression symptoms). Initial analyses were undertaken to examine the model assumptions for binary logistic regression. There were 93 cases that had standardized residuals that were greater than 3 which has been suggested previously as a cut-point to identify outliers (53). Further diagnostic analyses showed that among these cases, seven were having a large influence on the results and so these cases were removed from the final analysis. In line with a recent study (54) the independence of errors was assessed using the ratio of the deviance and Pearson distribution statistics to the degrees of freedom of the model. As both values were less than 1 (deviance: 0.46, Pearson: 0.95) this model assumption was not violated (54, 55). Multicollinearity was examined using the variance inflation factor (VIF). The highest VIF value was 3.1 which is below the value of 5 to 10 which has been suggested as an indicator of multicollinearity (56). Finally, a Box-Tidwell transformation procedure (57) showed that the linearity of the logit assumption was satisfied. Separate sex- and age-stratified analyses were then performed using the same model building process as previously described. All analyses were adjusted for location.

The analyses were performed with SPSS version 24 and Stata version 14 (StataCorp, College Station, TX, USA), with results being presented as odds ratios (OR) with 95% confidence intervals (CI). The level of statistical significance was p <0.05 (two-tailed).

## 3 Results

After removing those individuals that responded ‘don't know' to the fruit and vegetable questions, cases with missing values and influential outliers, data were analyzed from 3,410 adults with a mean (SD) age of 53.6 (18.1) years (range 18**–**89 years). The sample consisted of more women than men (52.0% > 48.0%). Regarding their dietary behavior, 5.3% (*n* = 181) of the respondents reported that they had a low level of fruit and vegetable consumption. The sample characteristics stratified by fruit and vegetable consumption status are presented in Table 1. Significant differences were observed in the prevalence of low fruit and vegetable consumption across all of the variable categories except for BMI and problematic alcohol use. In particular, the loneliness score was significantly higher in adults with low fruit and vegetable consumption (5.5 > 4.6). Other characteristics associated with low fruit and vegetable consumption included male sex, being middle-aged, low education, being single, low household income, being a current smoker and depressive symptoms.

**Table 1:** Sample characteristics by low fruit and vegetable consumption status.

		**Fruit and vegetable consumption**		
	**Total** ***n*** **(%)**	**Not Low** ***n*** **(%)**	**Low** ***n*** **(%)**	***p*** **-value**
Loneliness [M (SD)]	4.6 (1.6)	4.6 (1.6)	5.5 (2.0)	<0.001
	3,410 (100)	3,229 (94.7)	181 (5.3)	
**Sex**				<0.001
Male	1,638 (48.0)	1,509 (92.1)	129 (7.9)	
Female	1,772 (52.0)	1,720 (97.1)	52 (2.9)	
**Age**				<0.001
18–34	610 (17.9)	576 (94.4)	34 (5.6)	
35–59	1,396 (40.9)	1,292 (92.6)	104 (7.4)	
≥60	1,404 (41.2)	1,361 (96.9)	43 (3.1)	
**Education**				0.001
Higher education	2,169 (63.6)	2,075 (95.7)	94 (4.3)	
<Higher education	1,241 (36.4)	1,154 (93.0)	87 (7.0)	
**Marital status**				<0.001
Married/cohabiting	2,127 (62.4)	2,069 (97.3)	58 (2.7)	
Single (never married)	894 (26.2)	794 (88.8)	100 (11.2)	
Divorced/widowed	389 (11.4)	366 (94.1)	23 (5.9)	
**Household income (yen)**				0.001
4 <10 million	1,333 (39.1)	1,271 (95.3)	62 (4.7)	
≥ 10 million	252 (7.4)	248 (98.4)	4 (1.6)	
<4 million	1,083 (31.8)	1,005 (92.8)	78 (7.2)	
Missing	742 (21.8)	705 (95.0)	37 (5.0)	
**Medical conditions (number)**				0.029
0	2,139 (62.7)	2,016 (94.2)	123 (5.8)	
1	824 (24.2)	778 (94.4)	46 (5.6)	
≥2	447 (13.1)	435 (97.3)	12 (2.7)	
**Body mass index (BMI)**				0.749
18.5–24.9	2,364 (69.3)	2,245 (95.0)	119 (5.0)	
<18.5	420 (12.3)	396 (94.3)	24 (5.7)	
25.0–29.9	510 (15.0)	479 (93.9)	31 (6.1)	
≥ 30.0	116 (3.4)	109 (94.0)	7 (6.0)	
**Problematic alcohol use**				0.426
No	2,985 (87.5)	2,830 (94.8)	155 (5.2)	
Yes	425 (12.5)	399 (93.9)	26 (6.1)	
**Smoking status**				<0.001
Never smoker	2,105 (61.7)	2,020 (96.0)	85 (4.0)	
Former smoker	733 (21.5)	695 (94.8)	38 (5.2)	
Current smoker	572 (16.8)	514 (89.9)	58 (10.1)	
**Depressive symptoms**				<0.001
No	2,910 (85.3)	2,789 (95.8)	121 (4.2)	
Yes	500 (14.7)	440 (88.0)	60 (12.0)	

M, Mean; SD, Standard deviation.

**Figure 1:**
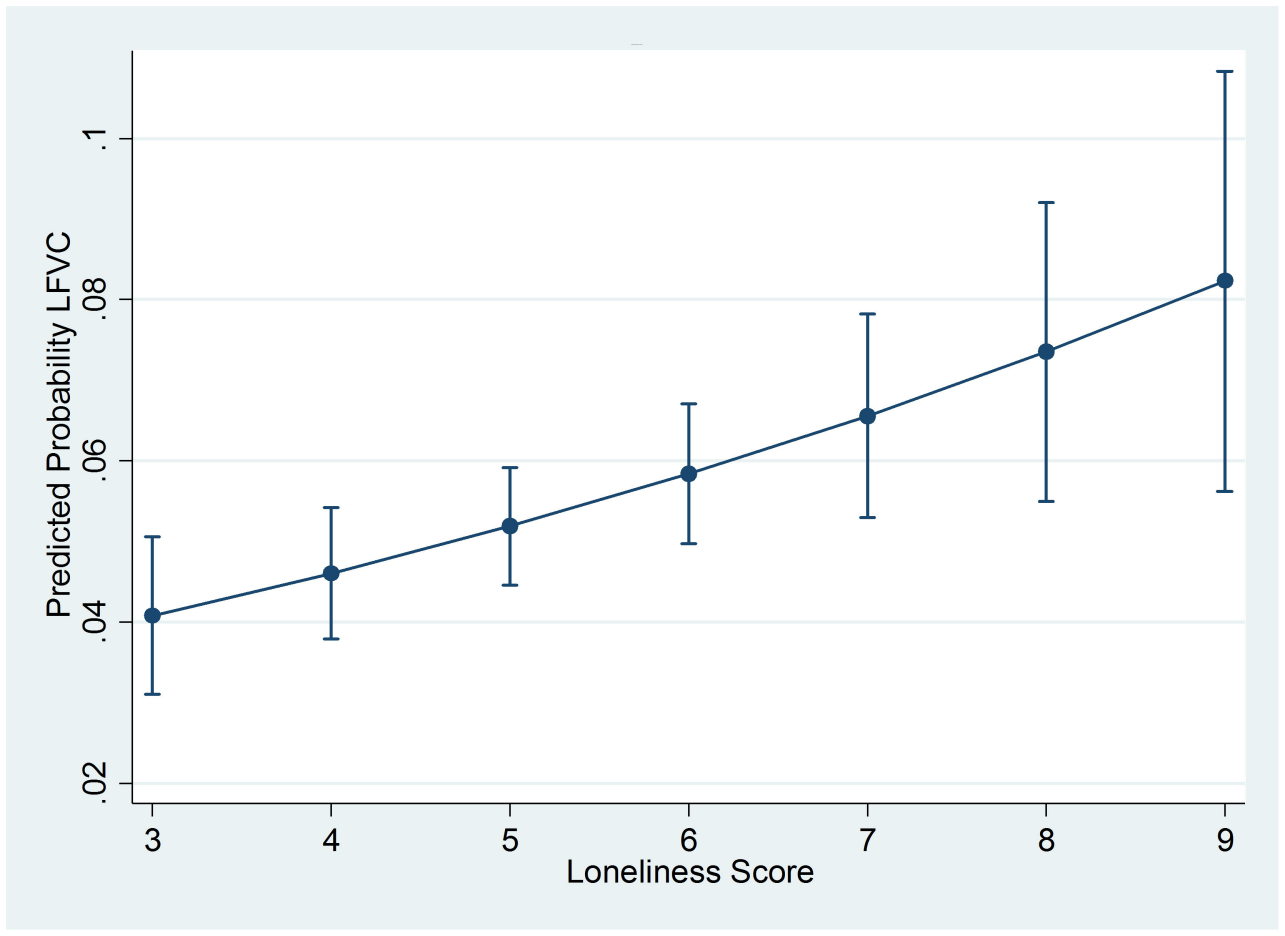
Predicted probabilities with 95% confidence intervals of low fruit and vegetable consumption (LFVC) by loneliness scores.

In a bivariate logistic regression analysis (Model 1) loneliness was associated with 33% higher odds for low fruit and vegetable consumption (OR: 1.33, 95%CI: 1.23-1.45) (Table 2), while the model had a pseudo R^2^ value of 0.04. Adjusting the analysis for sociodemographic variables (Model 2) slightly attenuated this association (OR: 1.21, 95%CI: 1.11-1.31). However, further adjustment for the physical health variables (Model 3), health-risk behaviors (Model 4), and depressive symptoms (Model 5) had little effect on the association as judged by the change in the ORs, while the pseudo R^2^ value increased by only 0.01 across each of these models. Thus, in the fully adjusted Model 5 loneliness continued to be associated with significantly higher odds for low fruit and vegetable consumption (OR: 1.14, 95%CI: 1.04-1.26), while the pseudo R^2^ value was 0.18. The association between the loneliness scores and low fruit and vegetable consumption is depicted in Figure 1 where it can be seen that the probability of low fruit and vegetable consumption increases as the loneliness score increases.

**Table 2:** The association between loneliness and low fruit and vegetable consumption in Japanese adults (*n* = 3,410).

	**Model 1**	**Model 2**	**Model 3**	**Model 4**	**Model 5**
	**OR (95%CI)**	**OR (95%CI)**	**OR (95%CI)**	**OR (95%CI)**	**OR (95%CI)**
Loneliness	1.33 (1.23–1.45)^***^	1.21 (1.11–1.31)^***^	1.21 (1.11–1.32)^***^	1.21 (1.11–1.32)^***^	1.14 (1.04–1.26)^**^
Sex (Female)		0.38 (0.27–0.54)^***^	0.36 (0.25–0.52)^***^	0.40 (0.28–0.58)^***^	0.39 (0.27–0.57)^***^
**Age**					
18–34		Ref.	Ref.	Ref.	Ref.
35–59		2.10 (1.37–3.24)^**^	2.19 (1.42–3.40)^***^	2.03 (1.30–3.15)^**^	2.09 (1.34–3.26)^**^
≥ 60		1.09 (0.64–1.86)	1.26 (0.71–2.22)	1.20 (0.68–2.12)	1.33 (0.75–2.36)
**Education**					
<Higher education		1.67 (1.22–2.29)^**^	1.68 (1.22–2.31)^**^	1.57 (1.14–2.17)^**^	1.56 (1.13–2.16)^**^
**Marital status**					
Married/cohabiting		Ref.	Ref.	Ref.	Ref.
Single (never married)		3.48 (2.36–5.14)^***^	3.54 (2.39–5.24)^***^	3.60 (2.43–5.33)^***^	3.50 (2.36–5.19)^***^
Divorced/widowed		2.57 (1.53–4.33)^***^	2.64 (1.56–4.45)^***^	2.60 (1.54–4.39)^***^	2.62 (1.54–4.43)^***^
**Household income (yen)**					
4 <10 million		Ref.	Ref.	Ref.	Ref.
≥ 10 million		0.32 (0.12–0.91)^*^	0.34 (0.12–0.95)^*^	0.34 (0.12–0.94)^*^	0.34 (0.12–0.96)^*^
<4 million		1.24 (0.85–1.81)	1.22 (0.83–1.79)	1.25 (0.85–1.83)	1.24 (0.84–1.82)
Missing data		0.94 (0.60–1.47)	0.93 (0.59–1.45)	0.98 (0.62–1.54)	1.00 (0.63–1.58)
**Medical conditions (number)**					
0			Ref.	Ref.	Ref.
1			1.07 (0.73–1.57)	1.04 (0.71–1.53)	1.02 (0.69–1.50)
≥2			0.53 (0.28–1.02)	0.51 (0.27–0.99)^*^	0.52 (0.27–1.00)
**Body mass index (BMI)**					
18.5–24.9			Ref.	Ref.	Ref.
<18.5			1.12 (0.69–1.81)	1.09 (0.67–1.77)	1.06 (0.65–1.73)
25.0–29.9			0.98 (0.64–1.51)	0.96 (0.62–1.48)	0.94 (0.61–1.45)
≥ 30.0			0.67 (0.29–1.53)	0.65 (0.28–1.49)	0.59 (0.25–1.35)
Problematic alcohol use				0.87 (0.55–1.38)	0.80 (0.50–1.28)
**Smoking status**					
Never smoker				Ref.	Ref.
Former smoker				1.30 (0.85–1.99)	1.27 (0.83–1.96)
Current smoker				1.94 (1.32–2.84)^**^	1.96 (1.33–2.88)^**^
Depressive symptoms					1.93 (1.32–2.83)^**^
Pseudo *R^2^* (Nagelkerke)	*0.04*	*0.15*	*0.16*	*0.17*	*0.18*

OR, Odds ratio; CI, Confidence interval; Ref, Reference category; ^***^p <0.001, ^**^p <0.01, ^*^p <0.05.

In Model 1 of the sex- and age-stratified analyses loneliness was associated with significantly higher odds for low fruit and vegetable consumption in every group except for young adults (age 18-34) (Table 3). However, further adjustment for the covariates resulted in differences between the groups. More specifically, in the fully adjusted Model 5 loneliness was associated with significantly higher odds for low fruit and vegetable consumption in women (OR: 1.32, 95%CI: 1.10–1.57) but not men (OR: 1.09, 95%CI: 0.97–1.21), and in middle-aged adults (OR: 1.14, 95%CI: 1.01–1.29) but not in younger (OR: 1.07, 95%CI: 0.86–1.33) or older adults (OR: 1.13, 95%CI: 0.91–1.40). However, caution should be exercised when it comes to interpreting these results given that the CIs were overlapping for all of the groups in the final model.

**Table 3:** Sex- and age-specific analyses of the association between loneliness and low fruit and vegetable consumption among Japanese adults.

	**Model 1**	**Model 2**	**Model 3**	**Model 4**	**Model 5**
	**OR (95%CI)**	**OR (95%CI)**	**OR (95%CI)**	**OR (95%CI)**	**OR (95%CI)**
**Sex**					
Men (*n* =1,638)					
Loneliness	1.22 (1.10–1.34)^***^	1.12 (1.01–1.24)^*^	1.12 (1.01–1.25)^*^	1.13 (1.02–1.25)^*^	1.09 (0.97–1.21)
Women (*n* =1,772)					
Loneliness	1.57 (1.35–1.82)^***^	1.44 (1.23–1.68)^***^	1.44 (1.23–1.68)^***^	1.45 (1.24–1.70)^***^	1.32 (1.10–1.57)^**^
**Age**					
18–34 (*n* = 610)					
Loneliness	1.17 (0.97–1.42)	1.13 (0.93–1.37)	1.13 (0.93–1.37)	1.15 (0.94–1.40)	1.07 (0.86–1.33)
35–59 (*n* = 1,396)					
Loneliness	1.28 (1.15–1.43)^***^	1.17 (1.05–1.31)^**^	1.17 (1.05–1.31)^**^	1.17 (1.05–1.31)^**^	1.14 (1.01–1.29)^*^
≥ 60 (*n* = 1,404)					
Loneliness	1.43 (1.20–1.70)^***^	1.30 (1.08–1.56)^**^	1.37 (1.13–1.65)^**^	1.35 (1.12–1.64)^**^	1.13 (0.91–1.40)

Model 1 examined the bivariate association between loneliness and low fruit and vegetable consumption; Model 2 was additionally adjusted for sex and age (as necessary), education, marital status and household income; Model 3 was additionally adjusted for medical conditions and BMI; Model 4 was additionally adjusted for problematic alcohol use and smoking status; Model 5 was additionally adjusted for depression symptoms.

OR, Odds ratio; CI, Confidence interval.

^*^p <0.05 ^**^p <0.01, ^***^p <0.001.

## 4 Discussion

This study used data from a sample of 3,410 adults from the Japanese general population that were obtained in an online survey in early 2023 to examine the association between loneliness and low fruit and vegetable consumption. Just over one in twenty adults (5.3%) were categorized as having low fruit and vegetable consumption (i.e., had not consumed fruit or vegetables in the past week). In a fully adjusted logistic regression analysis adults who were lonely had significantly higher odds for low fruit and vegetable consumption. Sex- and age-stratified analyses further indicated that this association may be stronger in women and middle-aged adults although confidence intervals overlapped for all of the sex and age groups in the fully adjusted analysis.

In this study just over 5% of adults consumed fruit and vegetables less than once a week. Comparing this figure with those from previous studies is complicated by a range of factors including the use of different definitions, methodologies and differences in study populations. However, there is some indication that this figure may be lower than in some other countries. For example, a recent study that used data collected from over 500,000 participants in China found that 40.5% consumed fruit less than weekly (39). Similarly, a study that used data from nine countries in the former Soviet Union that were collected in 2010 found that in Moldova 20% of the participants consumed both fruit and vegetables less than weekly, while in other countries over 20% of the respondents consumed fruit less than weekly while the corresponding figure for vegetables was over 10% (Belarus and Georgia) (46). However, that study also highlighted that the prevalence of inadequate fruit and vegetable consumption can vary widely across countries as in Azerbaijan only 4.6% and 6.4% of the participants respectively consumed fruit or vegetables less than once a week (46). It is uncertain why the prevalence of inadequate fruit and vegetable consumption might be lower in Japan although it is possible that it might relate to differences in a variety of sociodemographic, psychosocial and behavioral factors that have been previously linked to low fruit and vegetable consumption (49).

Previous studies that have examined the association between loneliness and low fruit and vegetable consumption in general population samples have produced mixed results (19–21). Moreover, studies in population subgroups have also produced similar mixed results with some studies finding an association, while in other studies there is no significant association. For example, a study that used data from African American church members found that individuals with high physical activity and high fruit and vegetable intake had reduced odds for loneliness (25). In contrast, a study that analyzed data from men and women aged 52 and above that were collected in the English Longitudinal Study of Aging found that loneliness was not associated with any differences in recommended daily fruit and vegetable intake over a 10-year period (24). It is unclear why the relation between loneliness and fruit and vegetable consumption has differed across studies. However, given the results from the current study and the fact that both loneliness (5) and low fruit and vegetable consumption (18) have been linked to worse health outcomes, then it suggests more research is now warranted on this association in different settings and among different population groups.

In a fully adjusted logistic regression analysis loneliness was significantly associated with low fruit and vegetable consumption in the total sample. As yet, there has been comparatively little focus specifically on the loneliness-low fruit and vegetable consumption relationship so it can only be speculated how these phenomena might be linked. For instance, it is possible that health literacy, which has been defined as “the degree to which individuals have the capacity to obtain, process, and understand basic health information and services needed to make appropriate health decisions” (58) might be relevant in this context given that an earlier study found that low health literacy was associated with both greater loneliness and insufficient fruit and vegetable consumption (59). Alternatively, other research has shown that social support from family/others may be an important factor in greater fruit and vegetable consumption (60). However, as there is also some evidence that levels of social support may be significantly lower in people who are lonely (51), it is possible that this might also link loneliness with lower levels of fruit and vegetable consumption. In addition, some research has also shown that higher levels of loneliness are associated with lower self-efficacy (61). This might also be of relevance given that an earlier review study found that self-efficacy regarding fruit and vegetable intake was strongly associated with its consumption (62).

Sex- and age-stratified analyses indicated that the association between loneliness and low fruit and vegetable consumption might be slightly stronger in women and middle-aged adults. It is uncertain whether these associations might be due to sex and/or age differences in the possible mechanisms outlined above or result from other factors. The finding for women is especially interesting given that low fruit and vegetable consumption was more prevalent in men in this study – itself a result that seemingly contradicts national data which show that the consumption of vegetables is lower in Japanese women than men (31). As regards specific mechanisms it has been suggested that loneliness might be more important for dysregulated eating in women than men (63) and that it might also be related to dietary restraint in women (64). Indeed, a recent study indicated that loneliness may be more strongly related to dieting in adulthood in women than men (65). Having said this, given the overlapping confidence intervals in the fully adjusted model across all of the population subgroups, more research is needed to determine if there are sex and/or age differences in the loneliness-low fruit and vegetable consumption association in Japan and the possible factors associated with them.

This study has notable strengths. It is the first large-scale study to examine the association between loneliness and low fruit and vegetable consumption in Japan. Also, to the best of our knowledge, it is the first study to examine if there are both sex and age differences in the association between loneliness and low fruit and vegetable consumption among adults in the general population. However, this study also has several limitations that should be mentioned. Information was collected on fruit and vegetable consumption with a single-item question that examined consumption during the past week. Collecting more detailed information over a longer period of time would have helped us to better specify the association. Further, as all the information was self-reported we cannot rule out the possibility that socially desirable responding may have been an issue. In addition, given that the respondents were selected from an online web panel it is possible that they were not fully representative of the underlying population in terms of their demographic characteristics and/or other factors (e.g. internet access). It is also possible that potentially important variables were missing from the analysis. For example, we had no information on urban or rural residence. As previous research has reported urban-rural differences in low fruit and vegetable consumption (46) and that loneliness scores can vary across levels of urbanization (66) it is possible that our results could have changed if this variable had been included. Finally, as this was a cross-sectional study we were not able to establish causal relations.

## 5 Conclusion

This study builds on previous literature by showing that there is an association between loneliness and low fruit and vegetable consumption among adults in the general population in Japan and extends previous work by further showing that this association may differ between population subgroups. In terms of future research, as cross-sectional studies have produced conflicting findings on the loneliness-fruit and vegetable consumption association, it is recommended that researchers should also undertake prospective studies to better determine the strength of this relationship. Moreover, it will also be important to establish the mechanisms linking loneliness with a poorer diet. Finally, as a recent study has indicated that the prevalence of loneliness in Japan may be comparatively high (29), then the results from the current study also underscore the potential importance and public health benefits of reducing loneliness in this setting.

## Data Availability

The raw data supporting the conclusions of this article will be made available by the authors, without undue reservation.
